# Histiocytic Sarcoma: Clinical Features and Outcomes of Patients Treated at a Tertiary Cancer Care Center

**DOI:** 10.7759/cureus.25814

**Published:** 2022-06-10

**Authors:** Deepa Susan Joy Philip, Amitha Sherief, Geetha Narayanan, Sreejith G Nair, Jayasudha AV

**Affiliations:** 1 Department of Medical Oncology, Regional Cancer Centre, Thiruvananthapuram, IND; 2 Department of Pathology, Regional Cancer Centre, Thiruvananthapuram, IND

**Keywords:** surgical resection, systemic chemotherapy, multifocal, unifocal, histiocytic sarcoma

## Abstract

Histiocytic sarcoma (HS) is an extremely rare histiocytic disorder of unknown etiology. It is not a true sarcoma and is named so, due to the pathological resemblance to mature histiocytes. The clinical presentation of HS is diverse and is related to the involved organs. Due to its aggressive nature, with poor prognosis and lack of a standard treatment regimen of choice, its diagnosis and management pose a challenge to the clinician. Limited literature is available on the management of this entity. Here, we report four patients with HS, diagnosed over 15 years in a tertiary cancer center, with varied clinical presentation, management, and outcomes. The first patient presented with a localized unresectable esophageal mass. He was treated with cyclophosphamide, doxorubicin, vincristine, and prednisone (CHOP) combination chemotherapy and attained complete remission. The second patient had a painless mass of the hand, treated with wide excision and adjuvant Radiotherapy. She is disease-free for the past 12 years. The third patient had presented with an anterior mediastinal mass. He had progressive disease on chemotherapy. The fourth patient had multifocal disease with generalized lymphadenopathy. She was treated with CHOP chemotherapy and is now disease-free at 13 months. To summarize, the patients with the localized resectable disease did well, with surgical excision and adjuvant radiotherapy, while patients with the multifocal disease did well on CHOP chemotherapy. The take-home message from this case series is - CHOP off whenever you can and if not give CHOP to chop off the disease.

## Introduction

Histiocytic sarcoma (HS) is an extremely rare histiocytic disorder of unknown etiology. It is usually diagnosed in the fourth and fifth decades of life with slight male preponderance. HS is not a true sarcoma. It is named HS because the neoplastic cells have a morphological and immunophenotypic resemblance to mature tissue histiocytes. It can affect nearly any organ system. The clinical presentation of HS is diverse and related to the involved organs. The most commonly involved organs are skin, soft tissue, and the gastrointestinal tract [1]. It presents with an aggressive clinical course with the majority of patients presenting with multi-system disease. It is diagnosed based on a detailed pathological evaluation of involved tissue, interpreted in the clinical context. Being a rare disease with a lack of prospective randomized evidence, there is no standard of care for the treatment of these patients. Localized disease is usually treated with surgery with or without adjuvant radiation. The multisystem disease is treated with chemotherapy. Due to its aggressive nature, poor prognosis, and, lack of a standard treatment regimen of choice, its management poses a challenge to the clinician.

Limited literature is available on the management of this entity. Here, we report four patients with HS, diagnosed over 15 years in a tertiary cancer center, with varied clinical presentation, management, and outcomes.

## Case presentation

Patient 1

**Figure 1:**
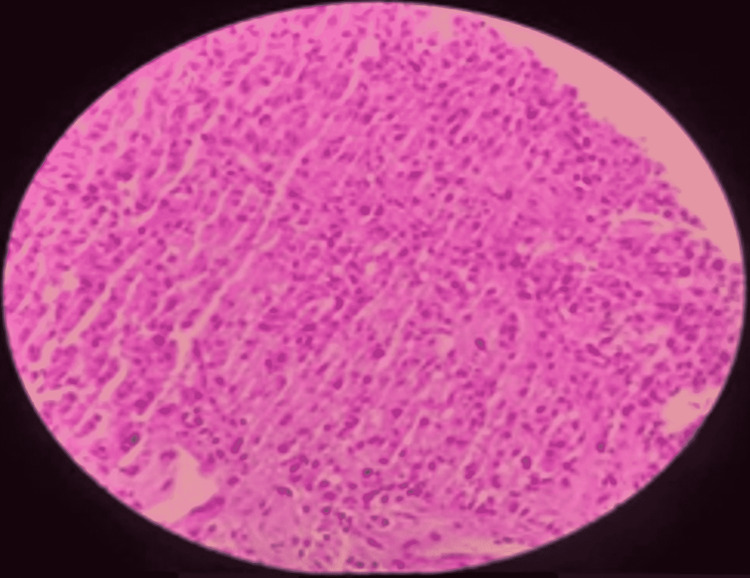
Histopathology and immunohistochemistry of patient 1 showing esophageal mass: upper GI endoscopy and biopsy. Histiocytoid tumor cells in an inflammatory background (low-power view).

**Figure 2:**
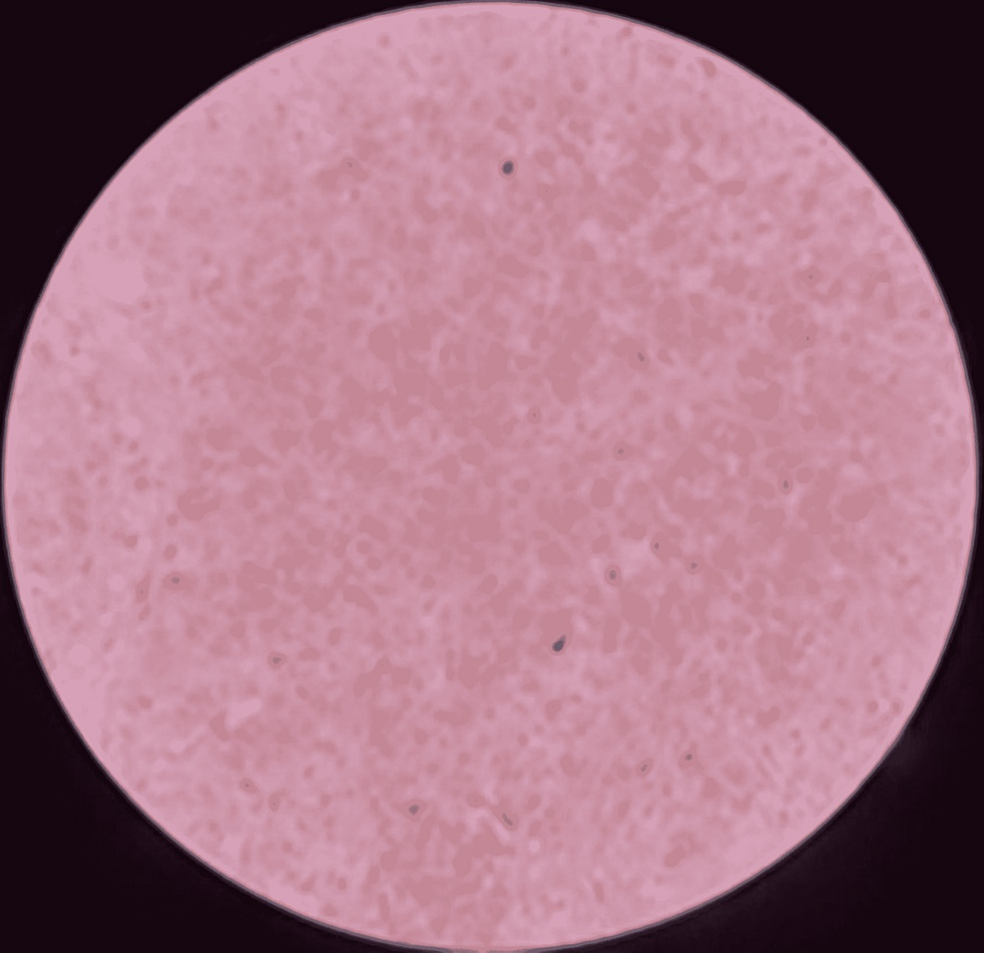
Histiocytic cells are CD68 positive (IHC, x400).

**Figure 3:**
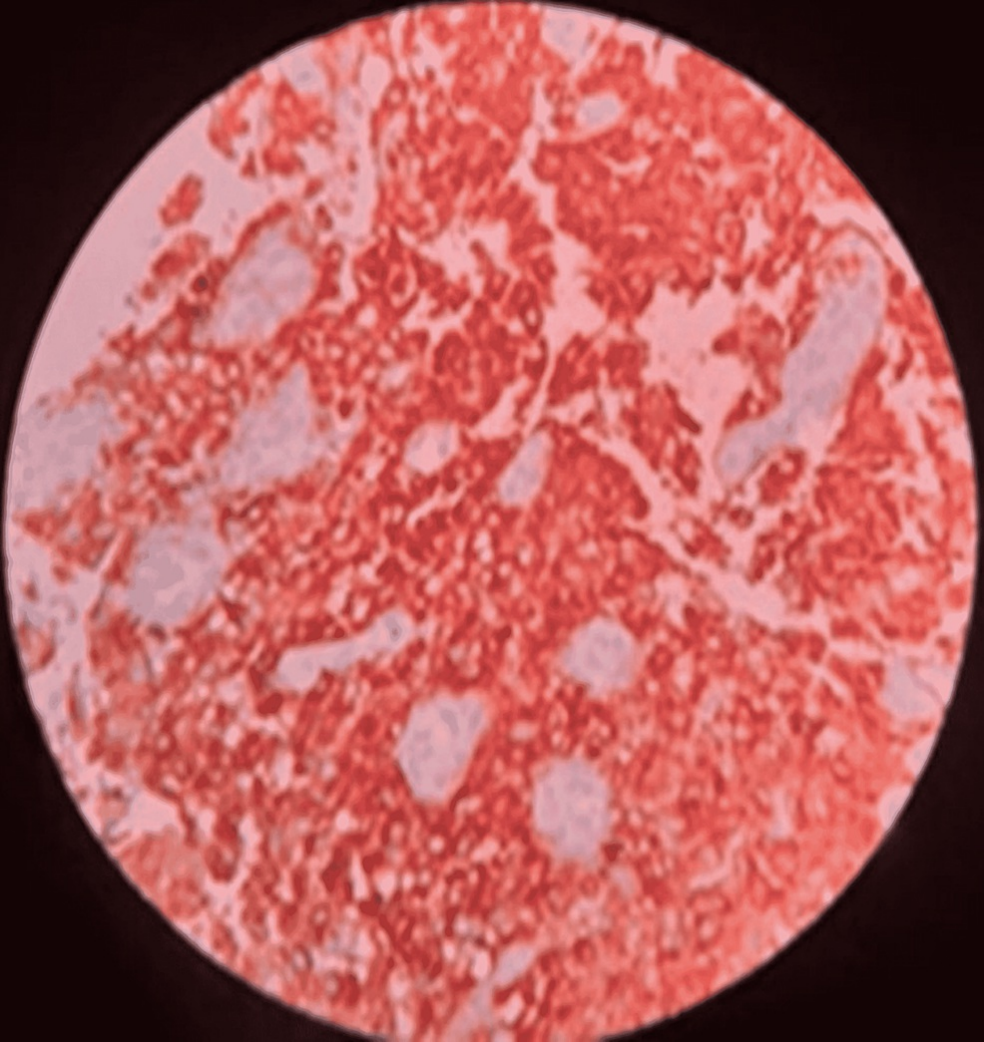
Histiocytic cells are CD163 positive (IHC, x400).

**Figure 4:**
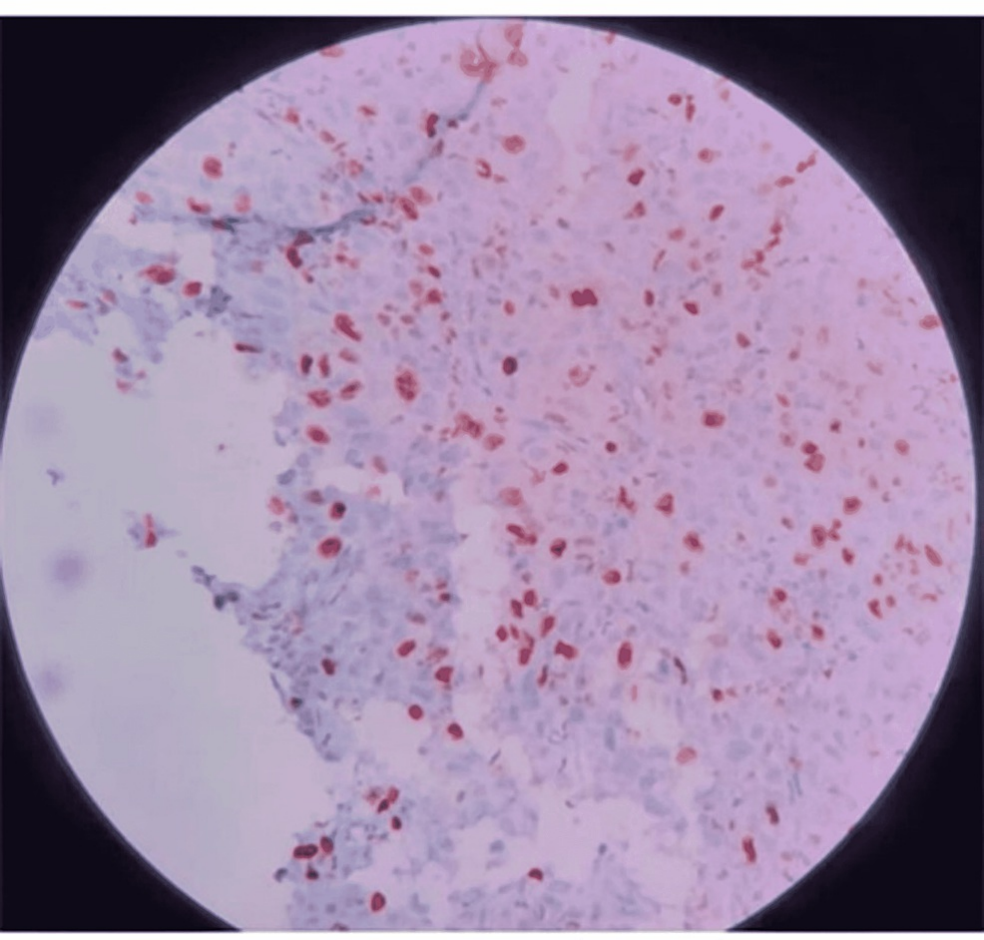
Histiocytic cells with high MIB%.

A 52-year-old gentleman presented with progressive dysphagia of two months duration. He was evaluated with an upper gastro-intestinal endoscopy, which showed an ulcero proliferative growth at 26 to 30 cm of esophagus. Contrast-enhanced CT scan thorax revealed an intramural soft tissue mass causing near-total luminal obstruction of the esophagus. This mass also caused extrinsic compression of the lower trachea and left main bronchus, resulting in the non-obstructive total collapse of the left lung. Posteriorly the mass was in contact with the arch of the aorta and descending aorta for more than 90 degrees. The patient underwent endoscopic biopsy, which showed Histiocytoid tumor cells, in a few nests of atypical cells having moderate to abundant eosinophilic cytoplasm and irregular nuclei in an inflammatory background. On immuno-histochemistry, the atypical cells were cytokeratin negative, CD163, CD68, and Vimentin positive. CD33 and LCA were weakly positive while Cytokeratin, P63, S100, Myeloperoxidase, CD1a, Langerin, CD34, CD20, and CD5 were negative, with a MIB1 labeling index of around 50%. The diagnosis of HS was confirmed (Figures 1-4).

 

 

 

**Figure 5:**
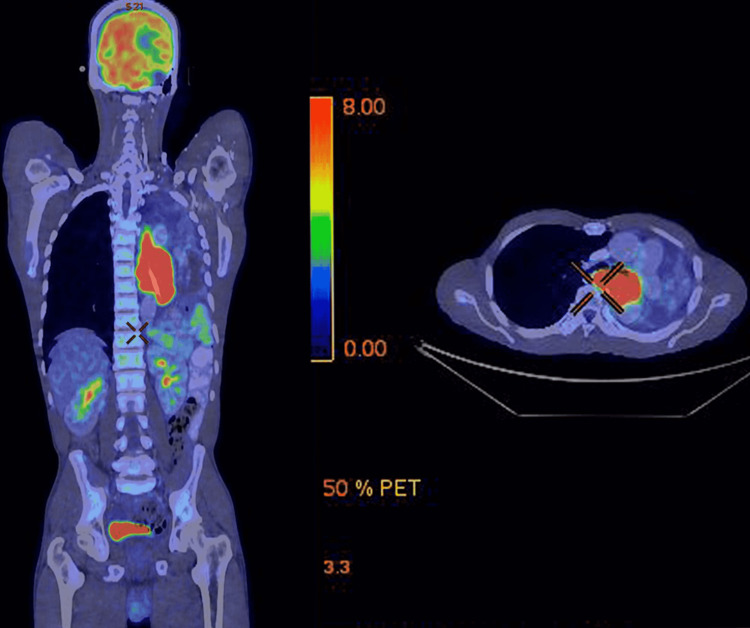
Pre-treatment PET-CT scan showing localized, unresectable lower thoracic esophageal mass

**Figure 6:**
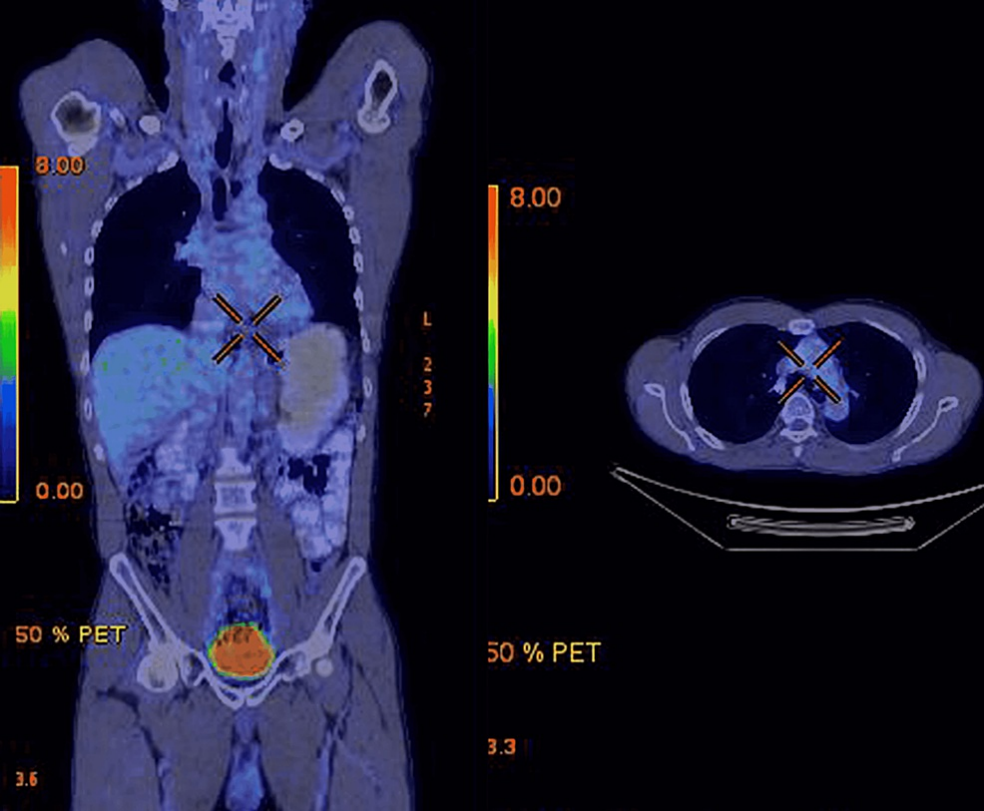
Post treatment PET-CT scan showing complete metabolic response

Staging workup with PET-CT evaluation revealed FDG avid, localized lower thoracic esophageal mass (Figure 5). Bone marrow examination was negative for malignant cells. The final diagnosis of localized HS of esophagus was made. Since the lesion was inoperable, he was started on systemic treatment. He received six cycles of chemotherapy with cyclophosphamide, doxorubicin, vincristine, and prednisone (CHOP). He tolerated chemotherapy well and post-chemotherapy evaluation with PET-CT showed complete metabolic response (Figure 6). Currently, he is alive and in complete remission at 12 months.

 

Patient 2

A 43-year-old lady presented with a painless swelling in the palmar aspect of left hand of one and half months duration. She presented to us after an unplanned excision of the swelling elsewhere. Histopathology of excised specimen was diagnostic of HS. The post-operative MRI of left hand showed presence of 2.2x2x2.8 cm residual lesion in the medial compartment of left hand. Metastatic work up was done with CECT thorax, abdomen and Bone marrow study which were negative. She underwent rewide excision of lesion. Intraoperative there was a 2x2 cm fibrous lesion involving hypothenar space of left hand infiltrating adjacent muscle and reaching deep up to carpal bones. She received adjuvant radiation 55Gy in 30 fractions. She is on follow-up and disease free for the last 12 years.

Patient 3

A 22-year-old boy presented with exertional dyspnea and cough. He was evaluated and found to have a bulky anterior mediastinal mass. He underwent partial resection of the mass outside and subsequently presented to us for further management. A repeat CECT thorax and abdomen was done, which showed residual mass of 4x4 cm in the anterior mediastinum. Histopathological review showed poorly differentiated malignant neoplasm. The serum AFP was 10,371 IU/mL. In view of the high tumor marker levels, the possibility of mediastinal germ cell tumor was considered. He received four cycles of BEP (Bleomycin, Etoposide, Cisplatin) chemotherapy; revaluation showed AFP in the normal range, but with radiological progression of residual mediastinal mass with multiple new lytic lesions in bone and multiple liver lesions. Due to the unusual nature of clinical presentation, the histopathological slides and blocks were reviewed with additional Immunohistochemistry. The tumor cells expressed CD68, CD163 with focal LCA, S100 positivity while negative for CK, CD23, CD1a. So, the final histopathological diagnosis was revised as “Histiocytic sarcoma associated with mediastinal germ cell tumour.” He was planned for six cycles of CHOP chemotherapy. Post four cycles of chemotherapy, PET-CT evaluation showed disease progression with a soft tissue mass lesion in lumbosacral region with impending spinal cord compression. Patient was given palliative radiotherapy to spine 20Gy in five fractions. His general condition deteriorated and he was deemed for best supportive care. He survived for 16 months since diagnosis.

Patient 4

A 61-year-old lady presented with incidentally detected left axillary swelling. Excision biopsy was suggestive of HS. Staging with PET-CT scan revealed non-bulky lymph nodes in cervical, axillary, and mediastinal areas with FDG non-avid lower esophageal thickening. She was treated with six cycles of CHOP chemotherapy. End of treatment evaluation with PET-CT revealed complete metabolic response and upper GI endoscopy was normal. At present, she is asymptomatic and on regular follow-up at 13 months.

**Table 1:** Clinical profile and treatment outcomes of patients with histiocytic sarcoma. CHOP - cyclophosphamide adriamycin oncovine prednisolone, CR - complete remission

Patient number	1	2	3	4
Age	52 yrs.	43 yrs.	22 yrs.	61 yrs.
Gender	Male	Female	Male	Female
Clinical presentation	Dysphagia	Hand swelling	Dyspnoea	Lt axillary swelling
Site	Oesophagus	Soft tissue	Mediastinal mass	Nodal
IHC	Positive markers: - LCA, CD163, CD68, CD33, Vimentin	Positive: LCA, KP-1, S100	Positive: CD68, CD163, LCA, S100	Positive: CD68 CD163
	Negative markers: - Cytokeratin,S100, P63 Myeloperoxidase, CD1a, Langerin CD34,CD20,CD5	Negative: CD30, ALK, HMB45	Negative: CD21, CD23, CD1a, CK, EMA	Negative: CK, MPO, CD34, CD20, CD5
Stage	Localized [unresectable]	Localized	Multifocal	Multifocal
Treatment	CHOP X 6	Surgery +RT	CHOP X 4	CHOP X 6
Response to treatment	CR	CR	PD	CR
Status at last follow-up	Alive and in CR at 12months	Alive and in CR at 144months [12yrs]	Death at 16 months	Alive and in CR at 13 months

The above table summarizes the clinical profile, pathology, treatment and outcomes of patients.

## Discussion

HS is an extremely rare non-Langerhan’s histiocytic disorder, constituting less than 1% of all hemato-lymphoid neoplasms [2]. HS has been associated with synchronous or metachronous hematologic malignancies and germ cell tumors. Due to its rarity and the possibility of overlapping morphological and immunophenotypical features, the diagnosis of HS is often challenging.

HS has histologic overlap with diverse mimics. So, the diagnosis is extremely challenging. The differential diagnosis is broad with diverse neoplasms having large epithelioid or pleomorphic cell patterns, including lymphomas (anaplastic large cell lymphoma, Hodgkin’s lymphoma), poorly differentiated carcinomas, melanoma, sarcomas (epithelioid sarcoma, epithelioid angiosarcoma), dendritic cell tumors (such as follicular dendritic cell sarcoma) and histiocytic diseases like Langerhans cell histiocytosis. Recognition of morphologic clues, and judicious application of immunohistochemical markers to confirm histiocytic differentiation and exclude mimics, is crucial.

The clinical presentation is varied depending on the organ of involvement. Most patients present with symptoms of unifocal or multifocal disease. Patients usually present with a palpable mass, compressive symptoms, or systemic symptoms. In the largest case series of 330 patients from the National Cancer Database (NCDB) reviewed by Kommalapati et al. [3], the most common sites of presentation were skin and connective tissue (41%), followed by lymph nodes (14%), gastrointestinal tract (12%), and hematopoietic system (9%). In our case series, patients had the disease in the gastrointestinal (esophagus), the soft tissue of the hand, mediastinal mass, and lymph nodes.

The diagnosis is based on histopathology with immunohistochemistry. The neoplastic cells are positive for histiocytic markers CD163, CD68, CD11c, and lysozyme and are negative for CK, T-cell, B-cell, and myeloid lineage markers with variable Ki-67.

Due to the rarity of the disease and lack of large prospective trials, there is no standard treatment regimen for HS. The stage of disease usually determines the choice of therapeutic options among systemic chemotherapy, surgery, and/or radiotherapy [4]. Patients with unifocal/localized disease are treated with surgical resection with or without radiotherapy. Patients with the multisystem, multifocal disease usually have a more aggressive clinical course and are treated with combination systemic chemotherapy regimens. Data regarding treatment are limited to small case series and case reports. Most clinicians use regimens designed for patients with clinically aggressive lymphomas. The most commonly used regimens are cyclophosphamide, doxorubicin, vincristine, prednisone (CHOP), ifosfamide, cisplatin, and etoposide (ICE), or doxorubicin, bleomycin, vinblastine, dacarbazine (ABVD).

In the largest case series by Kommalapati et al. [3], with 330 HS cases, the median overall survival (OS) was six months. Systemic chemotherapy alone was administered to 25% of the patients, while 22% had surgery alone. Patients with hematopoietic and reticuloendothelial system involvement were treated with chemotherapy alone or in combination with radiation. About 3% of the whole cohort underwent hematopoietic stem cell transplant. Improved survival was reported in patients with localized skin and connective tissue disease, treated with surgery with or without radiation. Adjuvant RT improved OS while adjuvant chemotherapy was not associated with similar benefits.

On the contrary, in patients with hematopoietic and reticuloendothelial system involvement, chemotherapy was associated with improved OS (median survival, 15 vs. 0.5 months; p < 0.0001). Thus, it has a guarded prognosis, with overall survival less than two years in treated cases and few months if untreated [5]. The possible prognostic factors include age, site of presentation, comorbidities, and therapy received. Patient 2 in our case series, with localized, resectable soft-tissue lesion did well with surgery and adjuvant radiotherapy and is on long-term follow-up. Patient 1, with localized but unresectable esophageal mass, and patient 4 with multifocal disease, were treated with CHOP chemotherapy, and have attained complete remission.

## Conclusions

HS is an extremely rare non-Langerhan’s histiocytic disorder with varied clinical presentations. Due to the very aggressive nature of the disease and lack of large prospective trials, there is no standard treatment regimen for HS. Patients with the localized resectable disease treated with surgery and adjuvant radiotherapy have good outcomes. Patients with multifocal disease are treated with combination systemic chemotherapy regimens. Targeted therapy may be the way forward in this orphan disease. To summarize, try to CHOP off whenever we can, if not CHOP off with CHOP.
